# A Survey on Prophylactic Corticosteroids Use in Stereotactic Radiosurgery Treatments From Ibero and Latin America Centers

**DOI:** 10.7759/cureus.34060

**Published:** 2023-01-22

**Authors:** Sergio Moreno-Jiménez, Daniel Álvarez-Guevara, Júlia Moscardini-Martelli, Carlos Barrios-Merino, Karen E Padilla-Leal, Ariadna Suárez-Venegas, Fabiola Flores-Vázquez

**Affiliations:** 1 Neurosurgery, National Institute of Neurology and Neurosurgery, Mexico City, MEX; 2 Neurosurgery, Neurological Center, American British Cowdray Medical Center, Mexico City, MEX; 3 Radiosurgery, National Institute of Neurology and Neurosurgery, Mexico City, MEX; 4 Radiotherapy, Centro Medico Nacional Siglo XXI, Mexico City, MEX; 5 Radiation Oncology, Cancer Center, American British Cowdray Medical Center, Mexico City, MEX

**Keywords:** radiation induced edema, radiation necrosis, prophylaxis, dexamethasone, corticosteroids, stereotactic radiosurgery

## Abstract

Introduction

Radiosurgery is a treatment in which a high dose of ionizing radiation is administered to a small field with high-precision techniques, and is a common treatment for tumors and other diagnoses. A typical complication is the development of radiation-induced edema that can progress to radiation necrosis in some cases. The administration of corticosteroids has been used empirically as a prophylaxis in patients who will be treated by stereotactic radiosurgery with intracranial tumors and other pathologies with the intention to prevent radiation-induced edema and or necrosis.

Objective

The aim of our study is to describe the actual use of corticosteroids in hospitals that perform stereotactic radiosurgery treatments in Latin America and Spain through a survey applied to neurosurgeons and radiation oncologists and expose the implications of the results, as well as to analyze the available literature on it.

Methods

We designed a questionnaire of 15 items related to the use of corticosteroids as prophylaxis in patients who will be treated with radiosurgery. The questionnaire was answered by 121 Ibero-Latin Americans through Google Drive considering a database from the Iberolatinoamerican Radiosurgery Association.

Results

We found that the preference for the use of corticosteroids as prophylaxis for radiosurgery is associated with informal training in radiosurgery, and it was more used by radiation oncologists compared to neurosurgeons (p=0.023). Side effects can exceed the benefit of its use.

Conclusions

There is practically no literature on the use of corticosteroids as prophylaxis for radiation necrosis in stereotactic radiosurgery. This is a controversial inter- and intra-specialty issue, and its empirical use has a relatively high prevalence, making us reconsider the value of experience in a medical environment that should be fundamentally guided by evidence-based medicine.

## Introduction

Radiosurgery is a treatment in which a high dose of ionizing radiation is delivered to a small field of the brain with high-precision techniques, either stereotactic or guided by imaging. One of the risks of this treatment is the development of radiation-induced edema that can progress to radiation necrosis in some cases. Brain radiation necrosis can affect the functional capacity of the patient and, depending on the region where it occurs, can decrease the patient’s quality of life. The likelihood of this complication depends on deterministic and stochastic factors [1]. There are two types of edema, namely cytotoxic and vasogenic, the latter being the most important in patients with intracranial tumors and radiation necrosis. This is explained by the pathophysiology that is based on the activation of the immune system and inflammation that generates progressive vascular damage, increasing vascular permeability [2]. Radiation necrosis and edema are closely associated. Indeed, researchers have determined that the greater the edema surrounding the lesion, as observed with magnetic resonance imaging (MRI), the greater the positive predictive value that the observed changes are due to radiation necrosis [3,4].

Corticosteroids alone or in combination with biological drugs have been used to treat symptomatic radiation necrosis. However, the use of corticosteroids is controversial in many fields of medicine: their indications, mechanism(s) of action, acute versus chronic use, real benefits, and toxic effects have not been fully defined. In addition, radiation necrosis, and therefore its consequences, have become refractory to steroid treatment in 70% of patients [5].

The administration of corticosteroids has been used empirically as prophylaxis in patients who will be treated by stereotactic radiosurgery for intracranial tumors or other pathologies. The main indication is to reduce the risk of post-treatment edema, although we must consider that another indication may be to avoid acute side effects such as headache, nausea, and vomiting. In addition, none of the studies citing the use of corticosteroids as prophylaxis for radiosurgery have described the variables related to who makes the decisions to use it or not or to justify its use. We seek to describe the use of prophylactic corticosteroids on a routine basis in hospitals that perform stereotactic or imaging-guided radiosurgery treatments in Latin America and Spain and to analyze the literature on the subject. We describe the results of a survey administered to Ibero-American neurosurgeons and radiation oncologists and the implication of these results.

## Materials and methods

We designed a 15-item questionnaire related to the use of corticosteroids as a prophylactic treatment in patients who will undergo intracranial radiosurgery for different tumor and non-tumor pathologies. The invitation to participate was through the Ibero-American Data Base, which includes 248 neurosurgeons, radiation oncologists, and medical physicists from Ibero-American countries. The self-administered questionnaire was applied to a population of 121 Spanish-speaking medical specialists through an electronic form in Google Drive. The inclusion criterion was neurosurgeons or radiation oncologists who perform radiosurgery procedures in their work hospitals. In addition, we conducted a general review of the literature to try to understand whether the use of corticosteroids on the day of cranial radiosurgery treatment is justified.

Statistical analysis

We present categorical variables as frequencies and percentages. We used the chi-square test or Fisher’s exact test for inferential analysis. We used XLSTAT from Excel v. 16.69 for the analysis.

## Results

Of the 121 medical specialists surveyed, 86% (n = 104) were physicians with the specialty of radiation oncology while 14% (n = 17) of respondents were neurosurgeons. The overall results of the survey are shown in Table 1.

**Table 1:** General survey results OAR = Organ at risk

Question	Variable	N	%
Specialty	Radiation Oncology	104	85.95
	Neurosurgery	17	14.05
Nationality	Argentina	11	9.09
	Colombia	3	2.48
	Costa Rica	5	4.13
	Ecuador	3	2.48
	El Salvador	3	2.48
	Spain	34	3.31
	Mexico	80	66.12
	Peru	3	2.48
	Dominican Republic	2	1.65
	Venezuela	7	5.79
Country of work	Argentina	11	9.09
	Colombia	1	0.83
	Costa Rica	2	1.65
	Ecuador	5	4.13
	El Salvador	3	2.48
	Spain	3	2.48
	Mexico	5	4.13
	Peru	81	66.94
	Dominican Republic	2	1.65
	Venezuela	5	4.13
Health sector	Public hospital	36	29.75
	Private hospital	42	34.71
	Both	43	35.54
Training	University	46	38.02
	Non-university	55	45.45
	Without training	20	16.53
Corticosteroid premedication	Always	48	39.67
	Selected cases	57	47.11
	Never	16	13.22
Routine use of corticosteroids agreement	Yes	56	46.28
	No	54	44.63
	Not sure	11	9.09
Time of administration (same day)	Before treatment	72	68.57
	After treatment	33	31.43
Corticosteroid	Methylprednisolone	1	0.95
	Prednisone	24	22.86
	Dexamethasone	79	75.24
	Betamethasone	1	0.95
Reason for use	Scientific evidence	55	52.38
	Empiric evidence	50	47.62
Avoid radiation-induced edema	Yes	77	73.33
	No	28	26.67
Avoid OAR toxicity	Yes	11	10.48
	No	94	89.52
Avoid acute symptoms	Yes	63	60.00
	No	42	40.00
Lack of scientific evidence for its use (for not users)	Yes	15	93.75
	No	1	6.25
No benefit in my practice (for not users)	Yes	1	6.25
	No	15	93.75

The age range was 28 to 78 years, with an average of 41.9 years. In terms of work experience, 58.7% (n = 71) of respondents had less than one to five years of experience in the field of radiosurgery, 20.7% (n = 25) had five to 10 years of experience, 4.1% (n = 5) had 10 to 15 years of experience, 5.0% (n = 6) had 15 to 20 years, and 11.6% (n = 14) had 20 years or more. In terms of training, 38% (n = 46) of respondents had taken a formal university course in radiosurgery, 45.5% (n = 55) had training without university endorsement, and 16.5% (n = 20) mentioned not having been trained in this discipline. For those with training, 28.1% (n = 34) mentioned that the duration of their radiosurgery training was 12 months, 15.7% (n = 19) mentioned that its duration was six months, 9.1% (n = 11) had two months of training, and 5.8% (n = 7) had 24 months of training. Regarding the administration of any corticosteroids prior to radiosurgery, the most commonly used doses were: a single 8 mg dose of dexamethasone (36.4%, n= 44), 50 mg of prednisone (10.7%, n = 13), and 4 mg of betamethasone and 50 mg of methylprednisolone only one case each.

Inferential analysis

**Figure 1:**
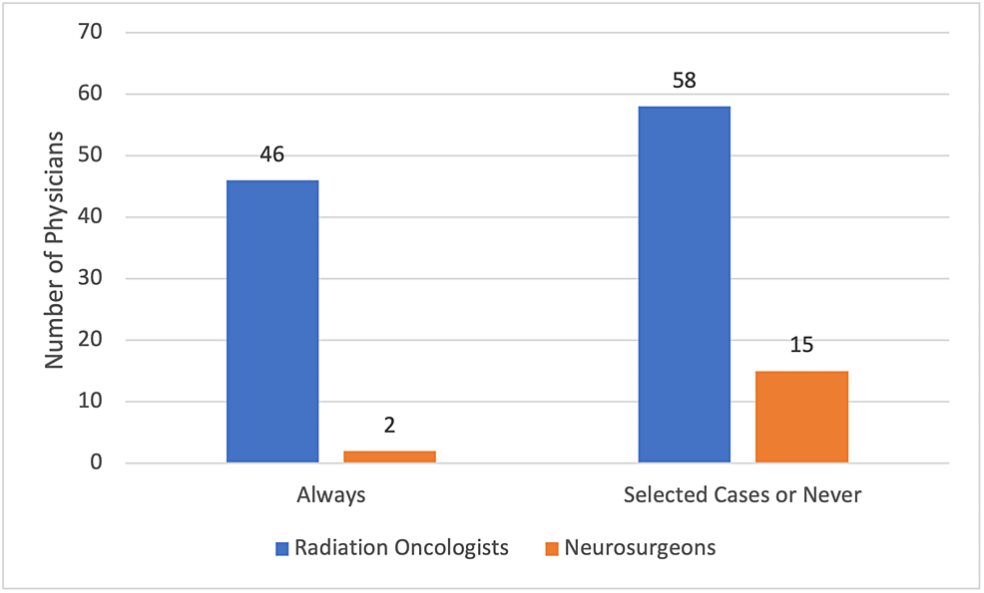
Use of corticosteroids by specialty Comparison of the use of prophylactic corticosteroids before stereotactic radiosurgery.

**Figure 2:**
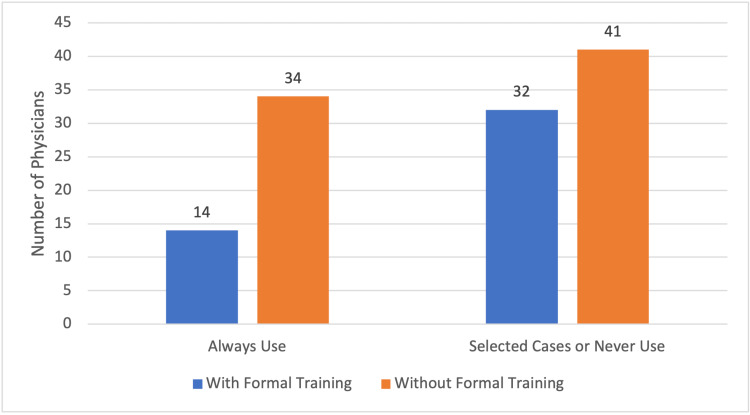
Usage of corticosteroids by training Comparison of physicians' with formal and informal training with respect to the use of prophylactic corticosteroids before stereotactic radiosurgery.

We found a significant difference between radiation oncologists and neurosurgeons regarding the prophylactic use of corticosteroids prior to stereotactic radiosurgery. Specifically, 46 radiation oncologists always use it and 58 only use it in selected cases or never, while only two neurosurgeons always use it and 15 only use it in selected cases or never (chi-square, p = 0.023). We found no significant difference with respect to specialists with formal or university training in radiosurgery compared with those who had no formal training. The results are shown in Figures 1-2 and Table 2.

**Table 2:** Comparison between radiation oncologists and neurosurgeons and between those with formal and no formal training

Variable	Use of corticosteroids N		
	Always	Not always	p-value
With formal training	14/46 (30%)	32/46 (70%)	0.151
Without formal training	34/75 (45%)	41/75 (55%)	
Radiation Oncology	46/104 (44%)	58/104 (56%)	0.023
Neurosurgery	2/17 (12%)	15/17 (88%)	

## Discussion

Radiosurgery is a treatment technique initiated in Sweden by Professor Lars Leksell, in which a high dose of ionizing radiation is administered using high-precision techniques [6]. The first treatment was performed in 1953 for a patient with trigeminal neuralgia; the radiation source was a Müller Mg Unit, an industrial X-ray machine of 280 kV. Since then, technological advances have allowed radiosurgery to evolve into a discipline with roots in both neurosurgery and radiation oncology, and patients have benefited from the collaboration of both specialists in conjunction with medical physicists. With the advancement of technology, more specific and precise techniques have also been developed in the field of radiosurgery; however, the complications that can occur after these procedures, although they occur at a low frequency, have also become evident [7,8]. Among the most prominent complications is radiation necrosis. Although the exact mechanism that generates it is imprecise, it is thought that the main trigger is endothelial vascular damage that causes apoptosis, astrocytic hypertrophy, and loss of blood vessels, generating tissue hypoxia and abnormal vascular proliferation and, finally, necrosis. There is a high correlation between radiation necrosis and the presence of edema surrounding the lesion, so radiation necrosis and a high degree of edema are normally associated. Corticosteroids as a treatment for radiation-induced edema are well known, as they have potent anti-inflammatory properties [9]. However, corticosteroids often have several associated adverse effects. In addition, administering corticosteroids on the day of treatment is debatable, considering that radiation-induced edema presents late. The use of various steroids has been described as premedication, although the reported doses are not necessarily equivalent [10]. Steroid dose equivalences are shown in Table 3.

**Table 3:** Steroid doses equivalences. Steroid dose equivalences. Modified from Walsh et al. [11] O = oral, IV = intravenous, IM = intramuscular

Corticosteroid	Relative anti-inflammatory potency	Relative mineralocorticoid potency	Plasma half-life (minutes)	Biological half-life (hours)	Route of administration	Equivalent anti-inflammatory dose (mg)
Dexamethasone	25 - 30	0	110 – 210	36 – 54	O, IV, IM	0.75
Prednisone	4	1	200 – 230	18 - 36	O	5
Methylprednisolone	5	0	78 – 188	18 - 36	O, IV	4
Betamethasone	25	0	300	36- 54	O, IV	0.6 – 0.75

Although there are some reports commenting on the use of corticosteroids as a treatment for peritumoral edema and radiation necrosis associated with radiosurgery, there is no evidence to support the benefit of using them as prophylaxis. In a clinical article, Sheehan et al. [12] described the routine administration of a single dose of intravenous dexamethasone unless the patient has a contraindication such as poorly controlled diabetes mellitus. Another study described the use of dexamethasone in patients operated on for metastasis resection and stereotactic radiosurgery [13]. The authors also mentioned extending corticosteroid treatment in patients who presented edema evidenced by MRI, worsening of the neurological deficit while weaning the patient off steroids, and leptomeningeal disease. They reported that complications associated with corticosteroid therapy occurred in 91.5% of patients. However, they did not make it clear at what point in therapy patients developed complications [14], mainly in the form of hyperglycemia [10].

In our study, 44.6% of the surveyed specialists reported disagreement with the routine use of corticosteroids as premedication in radiosurgery, 46.3% reported agreeing, and 9.1% reported not being sure. It should be noted that half of the respondents reported agreeing with the routine use of corticosteroids as premedication in radiosurgery.

Of the different variables analyzed, only specialty showed a significant difference: Radiation oncologists use prophylaxis more than neurosurgeons. Although we do not have a clear reason for this difference, we believe that it could be due to the more extensive training in radiobiology and the radiation-induced effects of the former. Of the respondents, 41.0% reported that the main reason they agreed with its use was because of their empirical experience. It is notable that there is a greater inclination of radiation oncologists to use corticosteroids, although there is a bias considering the limited sample of neurosurgeons surveyed.

Our results support the fact that the use of prophylaxis has been passed from generation to generation and is based primarily on an empirical decision. This fact makes us reconsider the value of experience and the “empirical” criterion in the use of prophylactic corticosteroids. Of the respondents who had formal training in radiosurgery, 30.6% answered that they always used prophylaxis while 52.0% of those who did not have training used it. The greater than 20% difference between these groups makes us think that formal training has some influence on the decision of respondents not to always use corticosteroids.

The most frequent reason why respondents considered that the use of corticosteroids is indicated is to avoid the complication of edema after radiosurgery (73.3%). When considering the relatively high frequency of cases in which post-radiosurgery edema is reported [10], we can understand why prophylactic corticosteroid treatment was the most chosen option. However, the frequency of complications from the use of corticosteroids is extremely high (91.5%) and thus the side effects can exceed the benefits [10]. Moreover, of the respondents who answered yes, 29.0% worked in public institutions, 16.0% worked in private institutions, and 55.0% worked in both institutions. This also allows us to infer that within public institutions there is a greater tendency to use corticosteroids prophylactically in radiosurgery.

Corticosteroids, also called glucocorticoids, regulate various cellular functions such as homeostasis, metabolism, cognition, and inflammation [15,16]. Administration prior to neurosurgery has potential benefits, but how long therapy should continue after the procedure is still controversial [11]. Another point to consider is that the half-life of dexamethasone is 36 to 56 hours. There is still a lot of doubt whether its administration on the day of treatment would really decrease the risk of radiation-induced edema or radiation necrosis. Prospective clinical trial-type studies with long-term follow-ups are required to answer this question.

## Conclusions

The preference for the use of corticosteroids as prophylaxis for radiosurgery is associated with the specialty of radiation oncology. The absence of formal training in radiosurgery shows a trend for greater use of prophylaxis. Due to the lack of evidence of benefit from prophylactic use of corticosteroids on the day of radiosurgery, to decrease the risk of complications from the procedure, together with evidence of potential side effects from its administration, we suggest that the risk outweighs the benefit. To our knowledge, there is no literature that justifies the routine use of corticosteroid prophylaxis in radiosurgery, so it is a topic for future studies. A limitation of the study is not considering prophylactic use specifically to avoid acute side effects such as headache, nausea and vomiting which could be studied in later work.
